# Gene x Gene Interactions Highlight the Role of Incretin Resistance for Insulin Secretion

**DOI:** 10.3389/fendo.2019.00072

**Published:** 2019-02-21

**Authors:** Benjamin Assad Jaghutriz, Martin Heni, Stefan Zoltán Lutz, Louise Fritsche, Fausto Machicao, Harald Staiger, Andreas Peter, Hans-Ulrich Häring, Andreas Fritsche, Róbert Wagner

**Affiliations:** ^1^Institute for Diabetes Research and Metabolic Diseases of the Helmholtz Center Munich at the University of Tübingen, Tübingen, Germany; ^2^German Center for Diabetes Research (DZD e.V.), Tübingen, Germany; ^3^Division of Endocrinology, Diabetology, Angiology, Nephrology and Clinical Chemistry, Department of Internal Medicine, Eberhard Karls University Tübingen, Tübingen, Germany; ^4^Institute of Experimental Genetics, Helmholtz Center Munich, Neuherberg, Germany; ^5^Department of Pharmacy and Biochemistry, Institute of Pharmaceutical Sciences, Eberhard Karls University Tübingen, Tübingen, Germany

**Keywords:** gene x gene interactions, SNP x SNP interactions, *TCF7L2*, *WFS1*, incretin resistance, incretins, insulin secretion, hyperglycemic clamp

## Abstract

**Introduction:** Genetic polymorphisms in *TCF7L2* are the strongest common risk variants for type 2 diabetes mellitus (T2D). We and others have shown that genetic variation in *TCF7L2* and *WFS1* affect incretin-stimulated insulin secretion. A recent genome-wide association study discovered genetic variants associated with incretin levels. We hypothesized that these SNPs (single nucleotide polymorphisms) interact with the well-known *TCF7L2* variant rs7903146 on insulin secretion due to their incretin altering effect.

**Methods:** In this retrospective analysis, we used data from the cross-sectional TUEF-cohort (*n* = 2929) and a hyperglycemic clamp study using additional GLP-1 infusion at the end of the clamp (*n* = 76). Insulin secretion was measured by evaluating OGTT-derived indexes of insulin secretion and insulin/C-peptide levels during clamp. We genotyped rs7903146 in *TCF7L2*, rs10010131 in *WFS1*, and six SNPs associated with GLP-1 and GIP levels.

**Results:** One of the six incretin-associated SNPs, rs17681684 in *GLP2R*, exhibited significant SNP x SNP interactions with rs7903146 in *TCF7L2* on insulin secretion (*p* = 0.0024) after correction for multiple testing. Three further SNP‘s showed nominally significant interactions (*p* < 0.05). In the hyperglycemic clamp study, rs7903146 in *TCF7L2* also interacted with rs17681684 on AUC C-peptide during the GLP-1 stimulation phase, thereby replicating the above finding.

**Conclusion:** The findings exemplify the role of SNP x SNP interactions in the genetics of type 2 diabetes mellitus and corroborate the existence of clinically relevant differences in incretin sensitivity.

## Introduction

Genome wide association studies (GWAS) have discovered multiple variants associated with type 2 diabetes mellitus (1–3). The largest proportion of these variants associates with insulin secretion (1).

However, for a substantial fraction of these variants, the exact pathophysiology still remains elusive. Among these variants, polymorphisms in or around the *transcription factor 7-like 2* (*TCF7L2*) gene currently represent the strongest signals (4, 5). Carriers of the *TCF7L2* risk allele have a significantly increased risk to develop type 2 diabetes mellitus (4). This is due to impaired insulin secretion associated with the risk variant. Using hyperglycemic clamps with GLP-1 infusion (6), we have previously shown that this *TCF7L2*-implicated defect in insulin secretion specifically involves insulin secretion stimulated by incretin action (7, 8). Another diabetes-related variant in the locus *WFS1* was shown to comparably impact incretin-sensitivity of the beta cell (2, 9). Incretins such as gastric inhibitory peptide (GIP), glucagon-like peptide-1 (GLP-1), and glucagon-like peptide-2 (GLP-2) are peptide hormones released from the small intestine (10–12). Among incretins, particularly GLP-1 and GIP are key factors of diet induced stimulation of insulin secretion accounting for up to 70% of postprandial insulin secretion (13).

A recent GWAS identified six variants associated with levels of fasting GIP, 2-h post-challenge GIP and 2-h post-challenge GLP-1 (rs1800437 in *GIPR*, rs17681684 in *GLP2R*, rs150112597 in *HOXD1*, rs927332 in *F13A1*, and rs635634 in *ABO*, rs17683011 in *SLC5A1*) (11). Using these variants as instruments, we attempt to elaborate on the pathophysiology of known incretin-dependent diabetes variants. Specifically, we hypothesize that gene x gene interactions exist between incretin variants and *TCF7L2* as well as *WFS1* on insulin secretion.

## Materials and Methods

### Subjects

Two thousand nine hundred twenty-nine subjects of the cross sectional Tübingen Family Study (TUEF) were included in the present work. The ongoing TUEF study recruits individuals at risk for type 2 diabetes mellitus (positive family history, prior gestational diabetes or known glucose intolerance or overweight) who are metabolically characterized. There were 76 participants in a separate hyperglycemic clamp study who provided DNA samples for genotyping (7). Both studies conformed to the principles outlined in the Declaration of Helsinki. All subjects gave informed written consent. Subject characteristics for the OGTT and the clamp study are shown in Supplementary Tables 1A,B, respectively.

### OGTT and Laboratory Measurements

All participants underwent a 5-point oral glucose tolerance test (OGTT) with 75 g glucose. Blood samples were taken at fasting and after 30, 60, 90, 120 min. Plasma glucose was measured using a bedside glucose analyzer (glucose-oxidase method, Yellow SpringsInstruments, Yellow Springs, OH, USA). Plasma insulin, C-peptide as well as other variables were measured with commercial chemiluminescence assays for ADVIA Centaur (Siemens Healthcare Diagnostics, Eschborn, Germany).

To quantify insulin secretion, we used corrected insulin response (CIR) and AUC _Insulin(0−30)_/AUC _Glucose(0−30)_ (AUC_secretion_) since both indices showed the highest sensitivity to capture genetic insulin secretion effects in different studies (14, 15). The AUC for insulin and glucose were calculated with the trapezoid method. Insulin sensitivity was assessed according to the insulin sensitivity index (ISI) of Matsuda and de Fronzo (16).

### Hyperglycemic Clamp

Hyperglycemic clamps were carried out as described previously (6). In brief, hyperglycemia with 10 mmol/l blood glucose was achieved with a continuous 20% dextrose solution, and GLP-1 was administered continuously 2 h after start. Additionally, an arginine bolus was applied at 180 min (6). Blood samples were taken according to the aforementioned study (6). C-peptide and insulin levels were measured at specific timepoints of the clamp (6).

### SNP Selection, Genotyping, and Genetic Risk Score

DNA was isolated from whole blood using a commercial kit (NucleoSpin, Macherey & Nagel, Dueren, Germany). Genotyping was carried out on the MassARRAY platform from Sequenom (Sequenom, San Diego, CA, USA). We genotyped the *TCF7L2* SNP rs7903146 as well as non-linked GWAS-derived variants associated with incretin levels, specifically rs17681684 (*GLP2R*), rs1800437 (*GIPR*), rs17683011 (*SLC5A1)*, rs150112957 (*HOXD1*), and rs927332 (*F13A1*) (11). One variant (rs150112597) had a very low minor allele frequency and was monoallelic in the smaller clamp dataset. The SNP rs10010131 in *WFS1* has been genotyped previously in a subset of 1,473 subjects. Allele distributions, minor allele frequencies and *p*-values for Hardy-Weinberg equilibrium are shown in Supplementary Table 2. We also calculated a weighted genetic risk score associated with post-challenge incretin levels derived from the work of Almgren et al. (11). Specifically, for each study participant with complete data on all investigated incretin-level modulating SNPs (rs17681684 in *GLP2R*, rs1800437 in *GIPR*, rs150112957 in *HOXD1*, rs927332 in *F13A1*), the number of per-SNP risk alleles was multiplied by the SNP-specific effect size associated with altered post-challenge incretin levels according to Almgren et al. (11).

### Statistics

All statistical analyses were conducted in R (V3.4). For linear regression models, outcome variables were log-transformed to approximate normal distributions. In order to facilitate comparison of genetic effects for different outcomes, effect sizes are shown as standardized estimates (β), with outcomes normalized to a mean of 0 with standard deviations of 1. In modeling genotypes, we used an additive inheritance model in the larger OGTT-dataset, and a dominant model in data of the hyperglycemic clamp study. The models fitted on insulin secretion using the corrected insulin response (CIR) were adjusted for sex, age, age^2^, BMI, insulin sensitivity (Matsuda-index). In order to reduce type 1 error, Bonferroni correction was applied. Therefore, an actual *p*-value of < 0.0041 (0.05/12) was considered as statistically significant. A *p*-value of < 0.05 but ≥0.0041 was referred to as nominally significant. Subjects with missing data were excluded from the analysis.

## Results

### OGTT-Based Insulin Secretion Study

**Figure 1:**
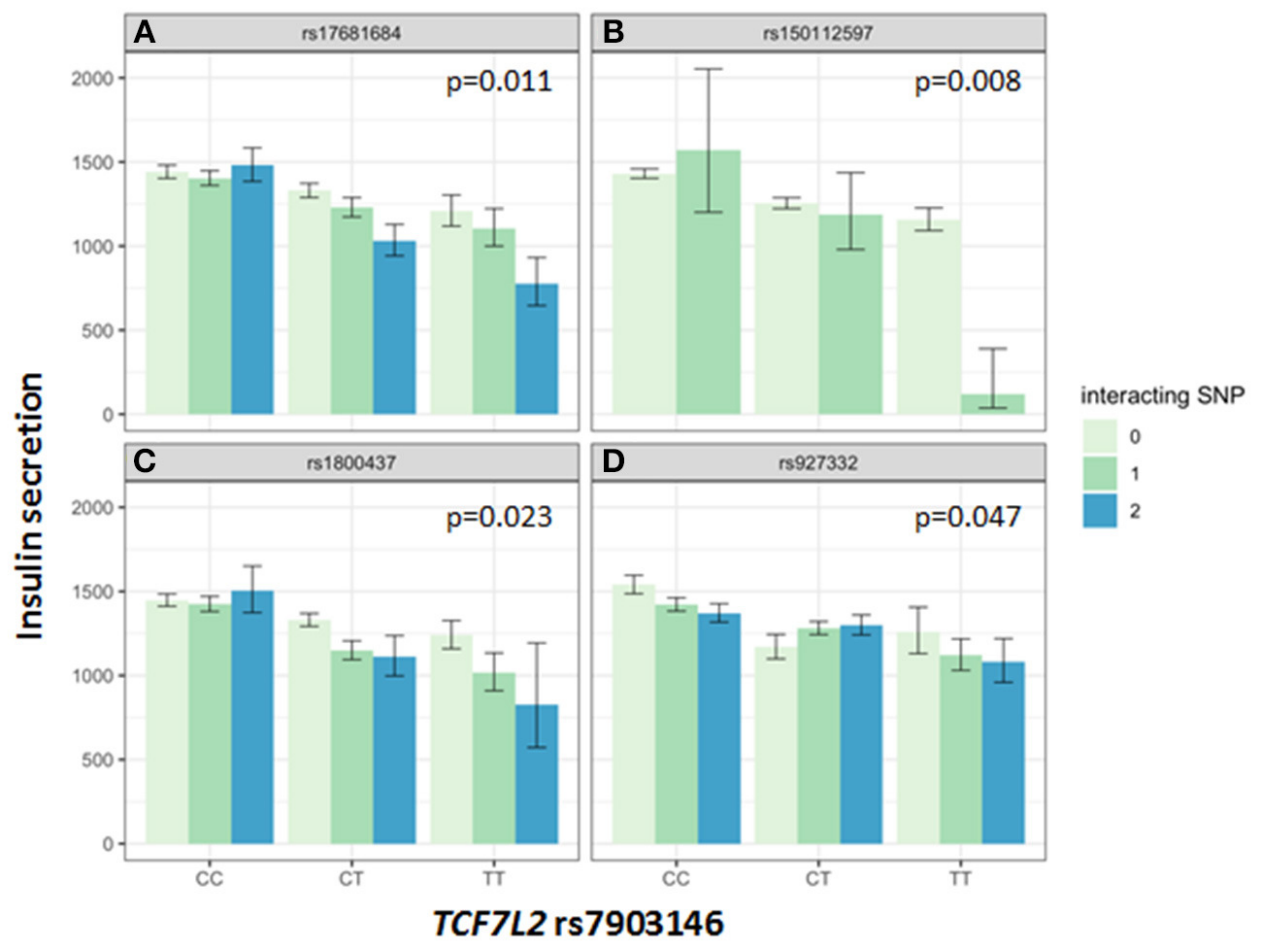
Impact of genotype combinations of *TCF7L2* and incretin modulating SNPs (rs17681684 in *GLP2R* **(A)**, rs150112957 in *HOXD1* **(B)**, rs1800437 in *GIPR* **(C)**, and rs927332 in *F13A1* **(D)** on insulin secretion (represented as CIR, geometric means with standard errors). Variants of rs7903146 in *TCF7L2* are depicted on the x-axis (CC, homozygous major allele; CT, heterozygous; TT, homozygous minor allele). Genotypes of incretin modulating SNPs (interacting with the *TCF7L2* variant) are represented by colors as minor allele counts (0, homozygous major allele; 1, heterozygous; 2, homozygous minor allele). The *p*-values refer to the interaction term of linear regression models adjusted for sex, age, age2, BMI, and insulin sensitivity (Matsuda-index).

**Figure 2:**
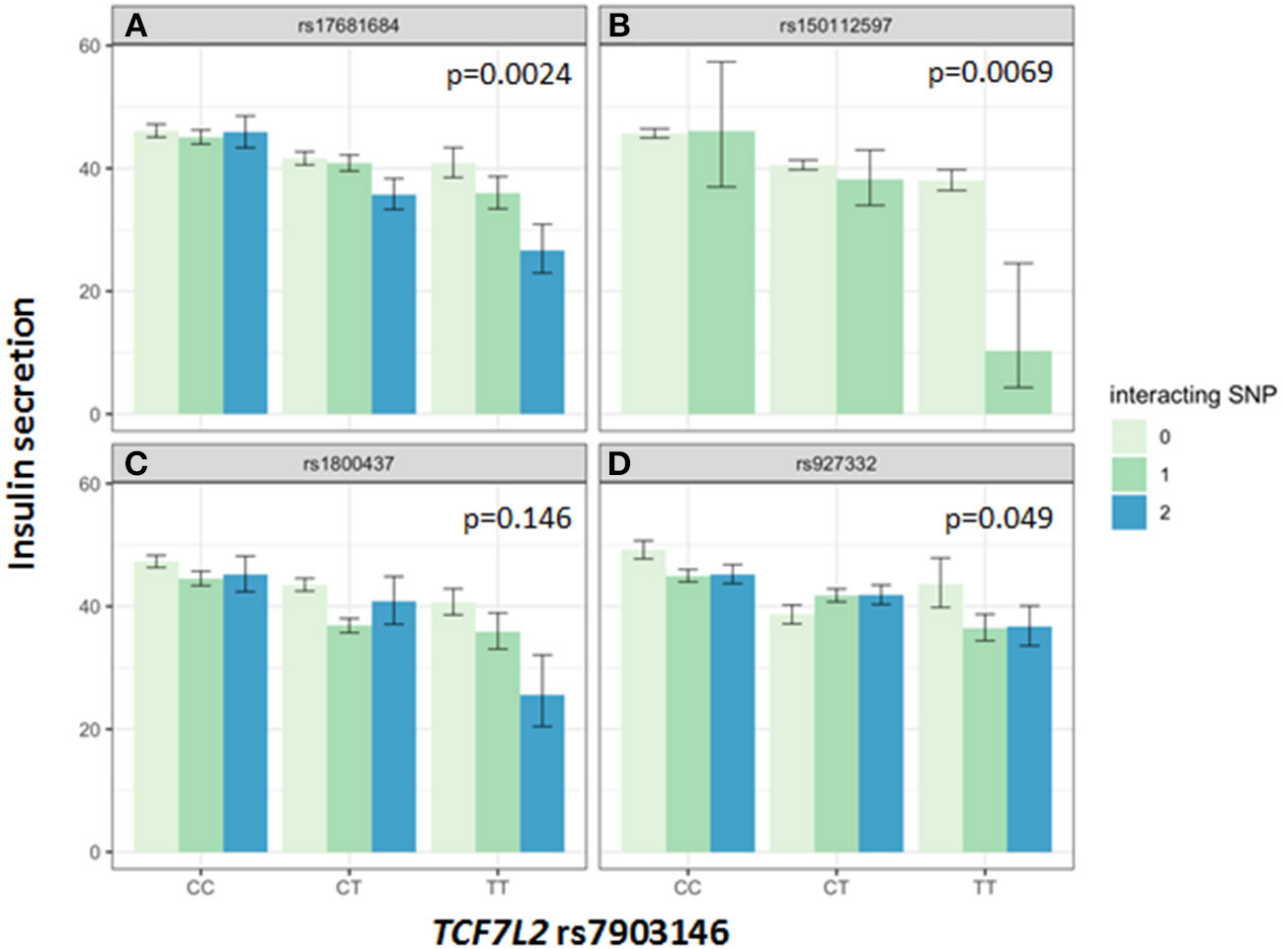
Impact of genotype combinations of *TCF7L2* and incretin modulating SNPs (rs17681684 in *GLP2R* **(A)**, rs150112957 in *HOXD1* **(B)**, rs1800437 in *GIPR* **(C)**, and rs927332 in *F13A1* **(D)** on insulin secretion (represented as AUC _Insulin(0−30)_/AUC _Glucose(0−30)_, geometric means with standard errors). Variants of rs7903146 in *TCF7L2* are depicted on the x-axis (CC, homozygous major allele; CT, heterozygous; TT, homozygous minor allele). Genotypes of incretin modulating SNPs (interacting with the TCF7L2 variant) are represented by colors as minor allele counts (0, homozygous major allele; 1, heterozygous; 2, homozygous minor allele). The *p*-values refer to the interaction term of linear regression models adjusted for sex, age, age2, BMI, and insulin sensitivity (Matsuda-index).

Association of all investigated SNP with insulin secretion (marginal effects of the main models, see below) are shown in Supplementary Tables 3, 4. We tested interactions between the *TCF7L2* variant rs7903146 and six described incretin-modulating SNPs in 2929 subjects who underwent OGTTs. From these six variants, four showed significant interactions with the *TCF7L2* variant on insulin secretion (Figures 1, 2). These SNPs were rs17681684 in *GLP2R* (interaction on CIR: β = −0.11 ± 0.043, *p* = 0.011; on AUC _Insulin(0−30)_/AUC _Glucose(0−30)_: β = −0.103 ± 0.034, *p* = 0.0024), rs150112597 in *HOXD1* (interaction on CIR: β = −0.782 ± 0.294, *p* = 0.008; on AUC _Insulin(0−30)_/AUC _Glucose(0−30)_: β = −0.626 ± 0.232, *p* = 0.0069), rs1800437 in *GIPR* (interaction on CIR: β = −0.104 ± 0.046, *p* = 0.023) and rs927332 in *F13A1* (interaction on CIR: β = 0.074 ± 0.37, *p* = 0.047; interaction on AUC _Insulin(0−30)_/AUC _Glucose(0−30)_: β = 0.057 ± 0.029, *p* = 0.049). Full results are shown in Supplementary Table 5.

Also, there was an interaction between one of the incretin level associated SNPs rs150112597 in *HOXD1* and rs10010131 in *WFS1* (interaction on CIR: β = 1.041 ± 0.438, *p* = 0.018; interaction on AUC _Insulin(0−30)_/AUC _Glucose(0−30)_: β = 0.877 ± 0.342, *p* = 0.01, also see Supplementary Figure 2, another gene known to modulate incretin-dependent insulin secretion. Full results for the WFS1 interactions are shown in Supplementary Table 6.

In order to assess the global effect of all investigated incretin-level modulating SNPs that interacted with *TCF7L2* on insulin secretion, we created a weighted risk score comprising these SNPs. This genetic risk score interacted with rs7903146 in *TCF7L2* on insulin secretion at a close-to genome-wide significance (*p* = 7.10^−6^), see Supplementary Figure 1.

### Hyperglycemic Clamp Based Insulin Secretion Study

To replicate these findings in a different cohort with other methods, we analyzed data of a cohort of participants undergoing hyperglycemic clamps with GLP-1 infusions. We found a nominally significant SNP x SNP interaction between the *TCF7L2* variant and the SNP rs17681684 in *GLPR2* (AUC _C−peptide(120−180)_: β = 0.384 ± 0.175, *p* = 0.031. An interaction between rs7903146 in *TCF7L2* and rs1800437 was also nominally significant in several phases of insulin secretion. Specifically, we found interactions for the first phase (AUC _C−peptide(0−10)_: β = −0.342 ± 0.131, *p* = 0.011), second phase (AUC _C−peptide(10−120):_ β = −0.32 ± 0.135, *p* = 0.02), and the GLP-1-stimulated phase (AUC _C−peptide(120−180)_: β = −0.308 ± 0.147, *p* = 0.04). Insulin and C-peptide levels during clamp in relation to the tested SNP-combinations are shown as Supplementary Figures 3, 4.

Regarding the interactions between rs10010131 in *WFS1* and the GLP-1-associated SNPs on insulin secretion during hyperglycemic clamps, the *WFS1* variant interacted nominally significant with rs927332 in *F13A1* on insulin secretion in all phases of the hyperglycemic clamp except for the first phase (AUC _Insulin(0−10)_: β = 0.367 ± 0.218, *p* = 0.097; AUC _Insulin(10−120)_: β = 0.564 ± 0.198 *p* = 0.0057; AUC _Insulin(120−180)_: β = 0.648 ± 0.257 *p* = 0.014).

## Discussion

Gene variants in *TCF7L2* are the strongest common genetic markers associated with T2D (1, 2). We have previously shown that GLP-1-stimulated insulin secretion is markedly reduced in *TCF7L2* risk-allele carriers (7). A similar impairment of incretin-dependent insulin secretion was found for another diabetes-risk variant in *WFS1* (9). After a recent work had identified six variants modulating GLP-1 and GIP levels, we utilized these variants as instruments to address the underlying pathophysiology of *TCF7L2* and *WFS1* (11). In this approach, we tested SNP x SNP interactions between variants modulating incretin levels and variants modulating incretin sensitivity. Our hypothesis implicated that an allele associated with low incretin levels in combination with the incretin resistance conveying allele of the *TCF7L2* variant would lead to a larger reduction of insulin secretion than a mere addition of both allele's effects.

For measures of insulin secretion, we used indices derived from an OGTT and, in a smaller cohort, from a hyperglycemic clamp with GLP-1 infusion. The results are summarized in Supplementary Figure 5. *TCF7L2* interacted significantly with one of six incretin-level variants in OGTT-derived insulin secretion data. There was a nominally significant association for further three variants (*GIPR, F13A1, HOXD1*). The SNP rs17681684 in *GLP2R* also showed an interaction with the *TCF7L2* variant in the hyperglycemic clamp, which replicated our main finding.

The key role of the *TCF7L2* in incretin sensitivity was corroborated by an interaction between a genetic risk score calculated from four incretin-level-modulating variants (*GLP2R, GIPR, F13A1, HOXD1*), and the *TCF7L2* variant. Incretins are particularly important for increasing insulin secretion after meals, as they depend on elevated glucose concentration, as physiologically present in the postprandial state. Therefore, OGTT-based indices vastly reflect the incretin effect. In the hyperglycemic clamp, one incretin-SNP showed a significant interaction with *TCF7L2* on C-peptide levels during GLP1-infusion. The first study describing features of lower insulin secretion in *TCF7L2* risk allele carriers showed a compromised insulin secretion during GLP-1 infusion (7). Lyssenko et al. indirectly demonstrated a reduced incretin effect in risk-allele carriers by comparing AUC insulin values from OGTT and intravenous glucose tolerance tests (17). The minor allele of rs17681684 in *GLP2R* is associated with lower fasting GIP and higher post-challenge GLP-1 levels (11). The interaction of this SNP with the *TCF7L2* variant shows that in case of a presence of homozygous minor alleles in both SNPs, the insulin secretion is compromised by 40% percent compared to carriers of the homozygous major alleles in both SNPs (see Figure 1). This finding suggests that in case of rs17681684, the post-challenge GLP-1 lowering effect predominates, counterweighing the increase of fasting GIP. This SNP is located in the *GLPR2* gene encoding the GLP-2 receptor, that is expressed in the gastrointestinal tract, hypothalamus, brain stem and lung (18). The ligand for this G-protein coupled receptor is the proglucagon-derived peptide GLP-2, which is released by L-cells of the small intestine (19). GLP-2 exerts multiple functions such as inducing intestinal epithelial growth and inhibiting of gastrointestinal motility (19, 20).

Further, there was a nominally significant interaction of the SNP rs1800437 in *GIPR* with the *TCF7L2* variant on insulin secretion in both the OGTT and the hyperglycemic clamp. In this case, the GIP and GLP-1 effects of the variant are also opposing. The minor allele of the GIPR variant is associated with lower fasting and post-challenge GIP, while there is a nominally significant association with increased fasting GLP-1 in the GWAS (11). We expect that a variant associated with higher GLP-1 levels would compensate for the decreased insulin secretion associated with the *TCF7L2* variant. Therefore, it is not probable that the interaction is driven by the GLP-1 effects of *GIPR*. Another possibility is that a decrease in GIP contributes to the interaction effect. Here, the effect direction of the *GIPR* variant on GIP levels would fit to our data. The constellation of the GIP-decreasing allele and the known risk allele of the *TCF7L2* variant leading to a more prominent decrease of insulin secretion could argue for this notion. However, we can probably differentiate chronic and acute effects of the *GIPR* variant in this case. Given that the effect of GIP on insulin secretion is diminished or absent during hyperglycemia (21), but our *TCF7L2* x *GIPR* interaction seems to impact insulin secretion during the whole hyperglycemic clamp, a role of an acute GIP effect is not probable. Instead, we speculate that a chronic effect of the *GIPR* variant e.g., on beta-cell mass (22, 23) interacts with the *TCF7L2* variant in this case, which could be related or not related to GLP-1 effects. This finding highlights the complex interplay of incretins and *TCF7L2*, and underlines the important role of both GLP-1 and GIP in the physiologic regulation of glycemia.

A limitation of the study is the relatively low sample size. To reduce type 1 error in interpretation of our data, we used multiple insulin secretion variables, a replication with a different method and Bonferroni-correction.

In summary, our data highlight a gene x gene interaction modulating incretin-stimulated insulin secretion. Such interactions can currently be only pinpointed by testing clear hypotheses. It remains a challenge to understand the pathology of T2D-associated variants such as rs7903146 in *TCF7L2*, because metabolic alterations are not efficiently detectable (24). However, it has been proposed that modeling gene x gene and gene x environment interactions could lead to a better understanding of the genetic architecture of T2D (25). Furthermore, our findings once again corroborate the existence of GLP-1 resistance which has clear clinical implications. For example, carriers of *TCF7L2* were shown to have a diminished response to an incretin-modulating dipeptidyl-peptidase IV inhibitor therapy (26). Genetically determined incretin resistance is also associated with a lower response to fiber-rich diet (27, 28). A better understanding of these interacting factors could aid individualized prevention and therapy of T2D.

## Author Contributions

BJ and RW analyzed the data and wrote the manuscript. MH, AF, HS, FM, AP, SL, and LF contributed to the interpretation of the data and edited the manuscript. MH and H-UH contributed to the study design and interpretation of data, and reviewed the manuscript.

### Conflict of Interest Statement

The authors declare that the research was conducted in the absence of any commercial or financial relationships that could be construed as a potential conflict of interest.

## Supplementary Material

Supplementary Figure 1 Regression lines depict the interaction of the genetic risk score for post-challenge incretin levels (lower numbers associated with lower post-challenge incretin levels, see methods) with rs7903146 in *TCF7L2* on insulin secretion (represented by CIR). The black line depicts the homozygous non-risk allele carriers, the yellow line the heterozygous and homozygous risk alle carriers in rs7903146. Data was adjusted for sex, age, age2, BMI, and insulin sensitivity (Matsuda-index). *P*-value < 10–6.

Supplementary Figure 2 Effects of the combination of genetic variants of the SNP rs10010131 in *WFS1* and the incretin modulating SNP rs150112957 in *HOXD1* on insulin secretion (represented as CIR, geometric means with standard errors). Variants of rs10010131 in *WFS1* are depicted on the x-axis (GG, homozygous major allele; GA, heterozygous; AA, homozygous minor allele). Genotypes of rs150112957 are represented in color bars as minor allele counts (0, homozygous major allele; 1, heterozygous allele). The *p*-values refer to the interaction term of the linear regression model adjusted for sex, age, age2, BMI, and insulin sensitivity (Matsuda-index).

Supplementary Figure 3 Insulin levels (insulin in pM, geometric means with standard errors) for combinations of the *TCF7L2* variant and the incretin-modulating SNP rs1800437 **(A)** and rs17681684 **(B)** during hyperglycemic clamp (10 mmol/l) with additional GLP-1 infusion and arginine stimulation. A dominant model is used. The presence of a *TCF7L2* risk allele is indicated by a green color, the presence of a *GIPR* **(A)***/GLP2R* **(B)** risk allele is indicated by a dotted line. Therefore, the green dotted line indicates both the presence of *TCF7L2* and *GIPR* **(A)**/*GLP2R* **(B)** risk allele (heterozygous and homozygous). The black continuous line indicates both the presence of *TCF7L2* and *GIPR* **(A)** /*GLP2R* **(B)** major allele.

Supplementary Figure 4 C-peptide levels (insulin in pM, geometric means with standard errors) for combinations of the *TCF7L2* variant and the incretin-modulating SNP rs1800437 **(A)** and rs17681684 **(B)** during hyperglycemic clamp (10 mmol/l) with additional GLP-1 infusion and arginine stimulation. A dominant model is used. The presence of a *TCF7L2* risk allele is indicated by a green color, the presence of a *GIPR* **(A)***/GLP2R* **(B)** risk allele is indicated by a dotted line. Therefore, the green dotted line indicates both the presence of *TCF7L2* and *GIPR* **(A)**/*GLP2R* **(B)** risk allele (heterozygous and homozygous). The black continuous line indicates both the presence of *TCF7L2* and *GIPR* **(A)**/*GLP2R* **(B)** major allele.

Supplementary Figure 5 Impact of the SNPs rs17681684, rs18000437, rs150112597, rs927332 on incretin levels (blue fields) according to Almgren et al. (11). Interaction between the incretin level modulating risk variants and incretin effect modulating risk variants rs7903146 in *TCF7L2* or rs10010131 in *WFS1* on insulin secretion determined in OGTT and hyperglycemic clamp (red fields). Effect direction is marked by triangles. Numbers after triangles represent standardized effect estimates. ^*^denotes nominal significant interactions on insulin secretion. ^**^denotes significant interactions on insulin secretion.

Supplementary Table 1 Baseline characteristics of the cohort.

Supplementary Table 2 Allele distributions, minor allele frequencies (MAF) and p-values of Hardy-Weinberg equilibria for the investigated variants.

Supplementary Table 3 Association of single SNPs with insulin secretion (CIR) in 2929 subjects adjusted BMI, sex, age, age^2^, insulin sensitivity (Matsuda-index).

Supplementary Table 4 Association of single SNPs with insulin secretion (AUC Insulin (0-30)/AUC Glucose (0-30)) in 2929 subjects adjusted BMI, sex, age, age^2^, insulin sensitivity (Matsuda-index).

Supplementary Table 5 Interaction of *TCF7L2* with incretin level related SNPs on multiple variables of insulin secretion (CIR, DI and (AUC Insulin (0-30)/AUC Glucose (0-30))) in 2929 subjects adjusted for BMI, sex, age, age^2^, insulin sensitivity (Matsuda-index).

Supplementary Table 6 Interaction of *WFS1* with incretin level related SNPs on multiple variables of insulin secretion (CIR, DI and (AUC Insulin (0-30)/AUC Glucose (0-30))) in 2929 subjects adjusted for BMI, sex, age, age^2^, insulin sensitivity (Matsuda-index).

## Abbreviations

CIR: corrected insulin response
DI: disposition index
GIP: gastric inhibitory peptide
GLP-1: glucagon-like peptide-1
GLP-2: glucagon-like peptide-2
ISI: insulin sensitivity index
OGTT: oral glucose tolerance test
SNP: single nucleotide polymorphism
TUEF: Tuebingen Family Study
T2D: type 2 diabetes mellitus.
