# Supplementary figures and images for: Gene x Gene Interactions Highlight the Role of Incretin Resistance for Insulin Secretion
Source: Front Endocrinol (Lausanne). 2019 Feb 21;10:72. doi: 10.3389/fendo.2019.00072 (PMC6393347; doi:10.3389/fendo.2019.00072)

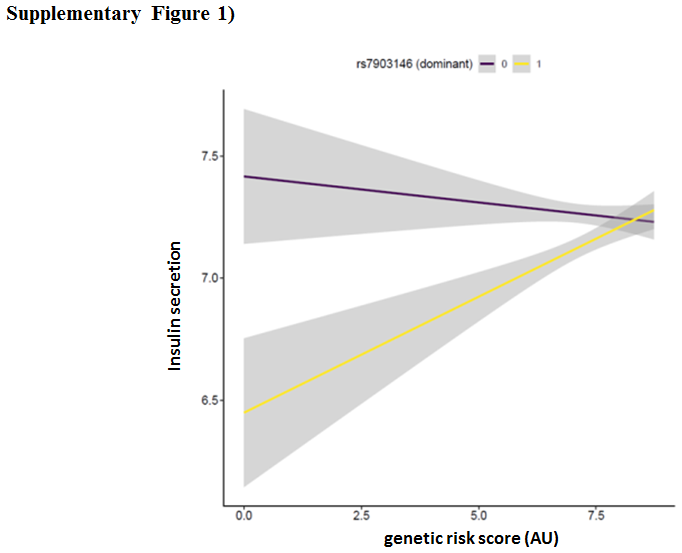

Supplement: Supplementary Figure 1 — Regression lines depict the interaction of the genetic risk score for post-challenge incretin levels (lower numbers associated with lower post-challenge incretin levels, see methods) with rs7903146 in TCF7L2 on insulin secretion (represented by CIR). The black line depicts the homozygous non-risk allele carriers, the yellow line the heterozygous and homozygous risk alle carriers in rs7903146. Data was adjusted for sex, age, age2, BMI, and insulin sensitivity (Matsuda-index). P-value < 10–6. [file Image_1.TIF]

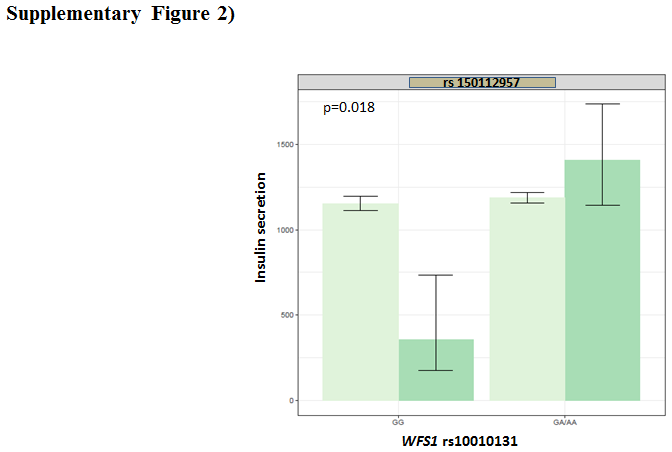

Supplement: Supplementary Figure 2 — Effects of the combination of genetic variants of the SNP rs10010131 in WFS1 and the incretin modulating SNP rs150112957 in HOXD1 on insulin secretion (represented as CIR, geometric means with standard errors). Variants of rs10010131 in WFS1 are depicted on the x-axis (GG, homozygous major allele; GA, heterozygous; AA, homozygous minor allele). Genotypes of rs150112957 are represented in color bars as minor allele counts (0, homozygous major allele; 1, heterozygous allele). The p-values refer to the interaction term of the linear regression model adjusted for sex, age, age2, BMI, and insulin sensitivity (Matsuda-index). [file Image_2.TIF]

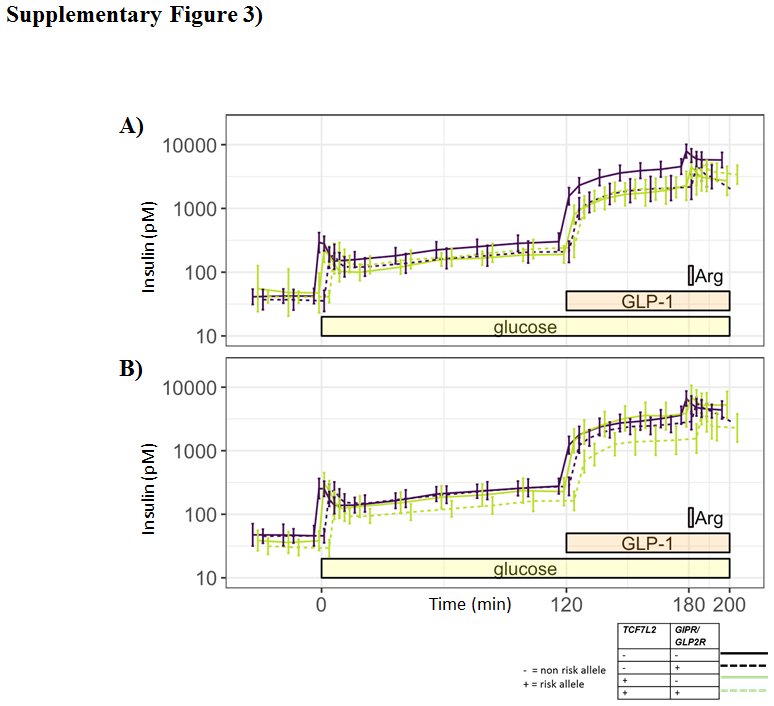

Supplement: Supplementary Figure 3 — Insulin levels (insulin in pM, geometric means with standard errors) for combinations of the TCF7L2 variant and the incretin-modulating SNP rs1800437 (A) and rs17681684 (B) during hyperglycemic clamp (10 mmol/l) with additional GLP-1 infusion and arginine stimulation. A dominant model is used. The presence of a TCF7L2 risk allele is indicated by a green color, the presence of a GIPR (A)/GLP2R (B) risk allele is indicated by a dotted line. Therefore, the green dotted line indicates both the presence of TCF7L2 and GIPR (A)/GLP2R (B) risk allele (heterozygous and homozygous). The black continuous line indicates both the presence of TCF7L2 and GIPR (A) /GLP2R (B) major allele. [file Image_3.TIF]

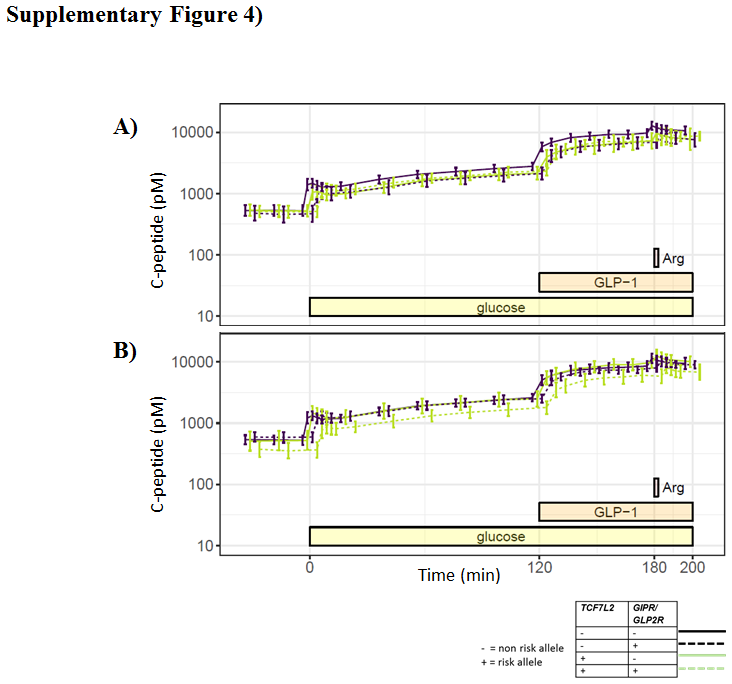

Supplement: Supplementary Figure 4 — C-peptide levels (insulin in pM, geometric means with standard errors) for combinations of the TCF7L2 variant and the incretin-modulating SNP rs1800437 (A) and rs17681684 (B) during hyperglycemic clamp (10 mmol/l) with additional GLP-1 infusion and arginine stimulation. A dominant model is used. The presence of a TCF7L2 risk allele is indicated by a green color, the presence of a GIPR (A)/GLP2R (B) risk allele is indicated by a dotted line. Therefore, the green dotted line indicates both the presence of TCF7L2 and GIPR (A)/GLP2R (B) risk allele (heterozygous and homozygous). The black continuous line indicates both the presence of TCF7L2 and GIPR (A)/GLP2R (B) major allele. [file Image_4.TIF]

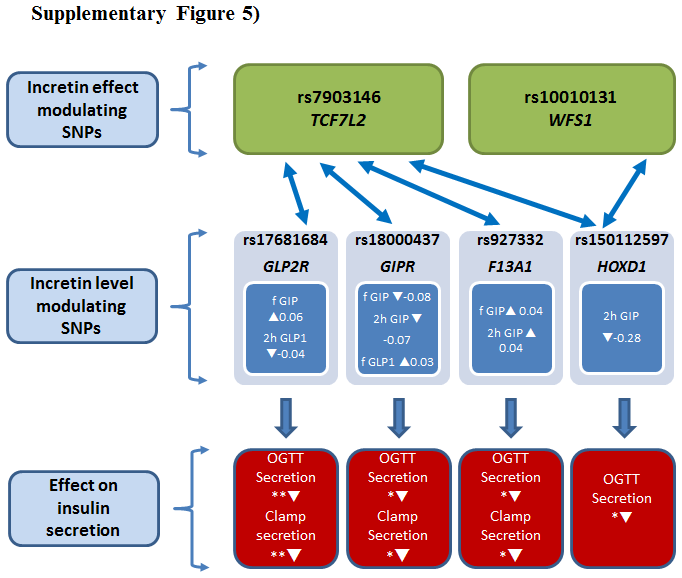

Supplement: Supplementary Figure 5 — Impact of the SNPs rs17681684, rs18000437, rs150112597, rs927332 on incretin levels (blue fields) according to Almgren et al. (11). Interaction between the incretin level modulating risk variants and incretin effect modulating risk variants rs7903146 in TCF7L2 or rs10010131 in WFS1 on insulin secretion determined in OGTT and hyperglycemic clamp (red fields). Effect direction is marked by triangles. Numbers after triangles represent standardized effect estimates. *denotes nominal significant interactions on insulin secretion. **denotes significant interactions on insulin secretion. [file Image_5.TIF]
