# Supplementary material for: Gene x Gene Interactions Highlight the Role of Incretin Resistance for Insulin Secretion
Source: Front Endocrinol (Lausanne). 2019 Feb 21;10:72. doi: 10.3389/fendo.2019.00072 (PMC6393347; doi:10.3389/fendo.2019.00072)
Supplement: Supplementary Table 1 — Baseline characteristics of the cohort. [file Data_Sheet_1.docx]

**Supplementary**

**Supplementary Table 1**

**A)**

Baseline characteristics of the whole study cohort. The median is shown for each continuous variable. Square brackets display the interquartile range [IQR].

| n | 2833 |
| --- | --- |
| Gender (w/m) | 1809/1024 |
| Age (years) | 43.00 [31.00, 55.00] |
| BMI (kg/m^2^) | 29.02 [24.49, 35.89] |
| Fasting glucose (mmol/l) | 5.22 [4.89, 5.67] |
| Glucose_120_ (mmol/l) | 6.33 [5.33, 7.67] |
| ISI-Matsuda (AU) | 9.38 [5.39, 15.57] |
| CIR (AU) | 1348.13 [811.69, 2230.81] |
| AUC _Insulin (0-30)_/AUC _Glucose (0-30)_ | 43.08 [28.08, 65.61] |

**B)**

Baseline characteristics of the sub-cohort, that underwent hyperglycemic clamp. The median is shown for each continuous variable. Brackets display the interquartile range [IQR].

| n | 76 |
| --- | --- |
| Gender (w/m) | 43/33 |
| Age (years) | 36.00 [26.00, 46.25] |
| BMI (kg/m^2^) | 24.48 [22.01, 26.94] |
| Fasting glucose (mmol/l) | 4.98 [4.54, 5.45] |
| Fasting insulin (mmol/l) | 35.20 [26.30, 55.35] |

# **Supplementary Table 2**

Allele distributions, minor allele frequencies (MAF) and p-values of Hardy-Weinberg equilibria for the investigated variants.

|  | **gene** | **major/minor allele** | **MAF** | **p-value** |
| --- | --- | --- | --- | --- |
| rs1800437 | *GIPR* | G/C | 0.22 | 0.14 |
| rs635634 | *ABO* | C/T | 0.23 | 0.14 |
| rs927332 | *F13A1* | T/C | 0.48 | 0.24 |
| rs17683430 | *SLC5A1* | G/A | 0.06 | 0.09 |
| rs17681684 | *GLP2R* | G/A | 0.29 | 0.25 |
| rs150112597 | *HOXD1* | G/C | 0.004 | 0.41 |
| rs7903146 | *TCF7L2* | C/T | 0.29 | 0.24 |
| rs10010131 | *WFS1* | G/A | 0.39 | 0.3 |

**Supplementary Table 3**

Association of single SNPs with insulin secretion (CIR) in 2929 subjects adjusted BMI, sex, age, age^2^, insulin sensitivity (Matsuda-index).

| **SNP** | **estimate** | **std. error** | **p-value** |
| --- | --- | --- | --- |
| rs7903146 | -0.1 | 0.022 | <0.00001 |
| rs17681684 | -0.05 | 0.023 | 0.025 |
| rs10010131 | 0.051 | 0.027 | 0.064 |
| rs1800437 | -0.042 | 0.024 | 0.083 |
| rs635634 | -0.037 | 0.024 | 0.12 |
| rs150112597 | -0.22 | 0.15 | 0.15 |
| rs17683430 | 0.037 | 0.042 | 0.38 |
| rs927332 | -0.012 | 0.02 | 0.54 |

**Supplementary Table 4**

Association of single SNPs with insulin secretion (AUC _Insulin (0-30)_/AUC _Glucose (0-30)_) in 2929 subjects adjusted BMI, sex, age, age^2^, insulin sensitivity (Matsuda-index).

| **SNP** | **estimate** | **std. error** | **p-value** |
| --- | --- | --- | --- |
| rs7903146 | -0.07 | 0.016 | 0.000012 |
| rs1800437 | -0.059 | 0.017 | 0.00082 |
| rs635634 | -0.039 | 0.017 | 0.023 |
| rs150112597 | -0.2 | 0.11 | 0.074 |
| rs17681684 | -0.022 | 0.016 | 0.17 |
| rs10010131 | 0.022 | 0.02 | 0.27 |
| rs927332 | -0.011 | 0.014 | 0.42 |
| rs17683430 | -0.0078 | 0.03 | 0.8 |

**Supplementary Table 5**

Interaction of *TCF7L2* with incretin level related SNPs on multiple variables of insulin secretion (CIR, DI and AUC _Insulin (0-30)_/AUC _Glucose (0-30)_) in 2929 subjects adjusted for BMI, sex, age, age^2^, insulin sensitivity (Matsuda-index).

| **variables of insulin secretion** | **interacting SNP** | **estimate** | **std. error** | **p-value** |
| --- | --- | --- | --- | --- |
| CIR | rs150112597 | -0.639 | 0.241 | 0.008 |
| CIR | rs17681684 | -0.09 | 0.035 | 0.01 |
| CIR | rs1800437 | -0.085 | 0.037 | 0.02 |
| CIR | rs927332 | 0.06 | 0.03 | 0.05 |
| CIR | rs17683430 | 0.052 | 0.068 | 0.4 |
| CIR | rs635634 | 0.018 | 0.038 | 0.6 |
| DI | rs150112597 | -0.665 | 0.253 | 0.009 |
| DI | rs17681684 | -0.084 | 0.037 | 0.02 |
| DI | rs17683430 | 0.106 | 0.071 | 0.1 |
| DI | rs927332 | 0.047 | 0.032 | 0.1 |
| DI | rs1800437 | -0.009 | 0.039 | 0.8 |
| DI | rs635634 | -0.001 | 0.04 | 1 |
| AUC _Insulin (0-30)_/AUC _Glucose (0-30)_ | rs17681684 | -0.066 | 0.022 | 0.002 |
| AUC _Insulin (0-30)_/AUC _Glucose (0-30)_ | rs150112597 | -0.401 | 0.148 | 0.007 |
| AUC _Insulin (0-30)_/AUC _Glucose (0-30)_ | rs927332 | 0.037 | 0.019 | 0.05 |
| AUC _Insulin (0-30)_/AUC _Glucose (0-30)_ | rs1800437 | -0.038 | 0.023 | 0.1 |
| AUC _Insulin (0-30)_/AUC _Glucose (0-30)_ | rs17683430 | 0.036 | 0.042 | 0.4 |
| AUC _Insulin (0-30)_/AUC _Glucose (0-30)_ | rs635634 | 0.001 | 0.023 | 1 |

**Supplementary Table 6**

Interaction of *WFS1* with incretin level related SNPs on multiple variables of insulin secretion (CIR, DI and AUC _Insulin (0-30)_/AUC _Glucose (0-30)_) in 2929 subjects adjusted for BMI, sex, age, age^2^, insulin sensitivity (Matsuda-index).

| **variables of insulin secretion** | **interacting SNP** | **estimate** | **std. error** | **p-value** |
| --- | --- | --- | --- | --- |
| CIR | rs150112597 | 1.041 | 0.438 | 0.018 |
| CIR | rs1800437 | 0.053 | 0.06 | 0.38 |
| CIR | rs17683430 | -0.06 | 0.094 | 0.52 |
| CIR | rs635634 | 0.036 | 0.06 | 0.54 |
| CIR | rs17681684 | 0.029 | 0.056 | 0.61 |
| CIR | rs927332 | -0.015 | 0.048 | 0.75 |
| DI | rs150112597 | 0.86 | 0.385 | 0.026 |
| DI | rs17683430 | -0.053 | 0.083 | 0.52 |
| DI | rs1800437 | 0.033 | 0.053 | 0.54 |
| DI | rs17681684 | 0.021 | 0.049 | 0.67 |
| DI | rs635634 | -0.006 | 0.052 | 0.9 |
| DI | rs927332 | -0.003 | 0.042 | 0.95 |
| AUC _Insulin (0-30)_/AUC _Glucose (0-30)_ | rs150112597 | 0.877 | 0.342 | 0.01 |
| AUC _Insulin (0-30)_/AUC _Glucose (0-30)_ | rs17681684 | 0.032 | 0.044 | 0.46 |
| AUC _Insulin (0-30)_/AUC _Glucose (0-30)_ | rs1800437 | 0.019 | 0.047 | 0.69 |
| AUC _Insulin (0-30)_/AUC _Glucose (0-30)_ | rs927332 | 0.014 | 0.037 | 0.71 |
| AUC _Insulin (0-30)_/AUC _Glucose (0-30)_ | rs635634 | -0.013 | 0.047 | 0.79 |
| AUC _Insulin (0-30)_/AUC _Glucose (0-30)_ | rs17683430 | -0.018 | 0.073 | 0.81 |
